# Earth's ionosphere D-region vertical electron density profile data during March 1-31, 2017 according to high-latitudinal Tumanny observatory partial reflection radar measurements and Polar Geophysical Institute's model

**DOI:** 10.1016/j.dib.2020.105848

**Published:** 2020-06-12

**Authors:** Aleksandr Gomonov, Roman Yurik, Oleg Zolotov

**Affiliations:** a Polar Geophysical Institute, Murmansk, Murmansk Region, 183010 Russia; b Murmansk Arctic State University, Murmansk, Murmansk Region, 183025 Russia

**Keywords:** Ionosphere D-Region, D-layer, electron density, vertical sounding, partial reflection radar, observations, modelling

## Abstract

The Earth's high-latitudinal D-Region ionosphere measurements are rarely performed continuously and with high temporal resolution. This data article provides the observed and modelled data on D-Region electron density variations during March 1-31, 2017 with 1-minute resolution. Both datasets share the same data-files’ structure and naming conventions. D-Region electron density observations were performed with the ground-based vertical sounding medium frequency (MF) partial reflection radar located at Tumanny observatory (69.0°N, 35.7°E). Modelling of D-Region ionosphere electron density variations were performed for the same period using Polar Geophysical Institute's theoretical model of quiet-time D-Region ionosphere. These datasets are valuable (a) to validate and cross-compare existing models of the D-Region ionosphere, (b) to verify and improve HF-band radio-waves propagation models, and (c) in investigations of the Earth's-ionosphere waveguide properties in dependence on the features of the lower part of the waveguide, i.e., the D-Region (often referenced to as the D-Layer). Both datasets are required to gain the reproducibility of results reported by Gomonov and co-authors [2019, doi: 10.1051/epjconf/201922403011].

Specifications table

**Table:** 

Subject	Earth and Planetary Sciences
Specific subject area	Earth's ionosphere D-Region observations and modelling
Type of data	Compressed (zipped) archive of standard ASCII plain text files
How data were acquired	Experimental (observed) data were derived from partial reflection radar measurements. Simulated data were calculated with Polar Geophysical Institute's D-Region model.
Data format	Raw, filtered, modelled
Parameters for data collection	The partial reflection radar is the ground-based device that operates on the regular basis at Tumanny observatory. The dataset in consideration was collected for geophysical conditions of March, 2013.
Description of data collection	Vertical electron density N_e_ profiles (1 min averages) were derived from Tumanny observatory partial reflection radar measurements during March 1-31, 2017. Simulated vertical electron density N_e_ profiles were calculated with Polar Geophysical Institute's D-Region model for geomagnetically quiet days of March, 2017.
Data source location	Institution: Polar Geophysical Institute (PGI) City/Town/Region: Murmansk, Murmansk Region Country: Russia Latitude and longitude for collected data: (69.0°N, 35.7°E)
Data accessibility	The dataset is provided with the article.
Related research article	A. Gomonov, R. Yurik, Yu. Shapovalova, S. Cherniakov, O. Ogloblina, Comparison of numerical simulations of the electron density in the D-region ionosphere with measurements of the radar of partial reflections, EPJ Web of Conferences, DOI: 10.1051/epjconf/201922403011, https://doi.org/10.1051/epjconf/201922403011[1]

## Value of the Data

• Continuous D-Region (commonly referred to as the D-Layer) observations are rarely performed and made available to public. The proposed dataset is a valuable deposit to available high-latitudinal Earth's ionosphere D-Region observations; 1-minute resolution of the data is also a significant feature of the proposed dataset.
• Both the observed and model datasets are valuable for validation and comparison of different models of the Earth's D-Region ionosphere. The data may be used to improve (calibrate) D-Region models or to assimilate the data in case of data assimilation models.
• The data may be used to validate and improve the HF-band radio-waves propagation models. It is also suitable for investigations of the Earth's-ionosphere waveguide properties in dependence on the features of the lower part of the waveguide (i.e., the D-Region).
• The following models (listed in alphabetical order) could be contrasted against the published with this paper datasets: (a) the Absorption by the D and E Region of HF signals with NORMAL Incidence (ABBYNormal) model [2]; (b) D-Region Absorption Prediction (D-RAP) model [3]; (c) International Reference Ionosphere (IRI) model [4]; (d) Sodankylä Ion Chemistry (SIC) model [5]; (e) Thermosphere Ionosphere Mesosphere Electrodynamics General Circulation (TIME-GCM) model [6]; (f) Whole Atmosphere Community Climate Model with D-region ion chemistry (WACCM-D) [7]. These datasets are also required to gain the reproducibility of reported by Gomonov and co-auth. [1] results.

## 1 Data Description

The proposed dataset contains the observed and modelled data on vertical electron density Ne profiles for the Earth's ionosphere D-Region during March 1-31, 2017. Electron density values are presented in units of [cm^-3^]; corresponding altitudes are counted from the Earth's surface and are provided in units of [km]. All timestamps are provided in UT (Universal Time). The time-step is 1 min in all cases. It corresponds to (a) 1 min averages for the observations and (b) instant snapshots for the model case.

The dataset is provided in form of compressed (zipped) archive that contains (a) ‘pr_radar’ directory, (b) ‘model’ directory, (c) ‘example.ipynb’ file as well as (d) ‘README’ file. The ‘README’ file contains short description of the dataset distribution. The Jupyter notebook (i.e., ‘example.ipynb’ file) provides code snippets for the data loading, processing, and plotting. The ‘pr_radar’ directory contains plain text human-readable ASCII data-files with electron density observations; one data-file per date. Data-files follow ‘neYYYYDDD’ naming convention, where ‘ne’ denotes an electron density Ne, ‘YYYY’ represents a year (four digits), and ‘DDD’ is a day of a year (three digits with leading zeroes when required). For example, March 1, 2017 should correspond to ‘ne2017060’ name of the file.

**Fig. 1:**
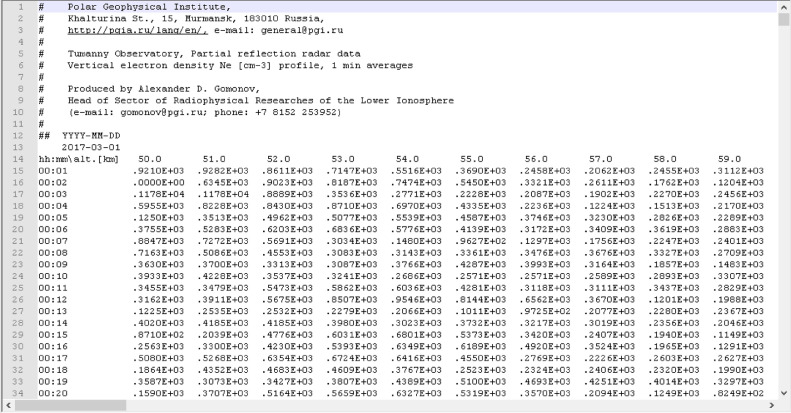
The scheme of the ASCII data-file format for partial reflection radar electron density Ne profiles. Header lines (escaped with the ‘#’ symbol) provide information on the data holder, instrumental facility, the data type, and the data producer. Next lines provide a data-label of the observations, and a space-separated electron density data-table. One line (row) corresponds to an altitudinal Ne profile, i.e., to a single moment in time.

**Fig. 2:**
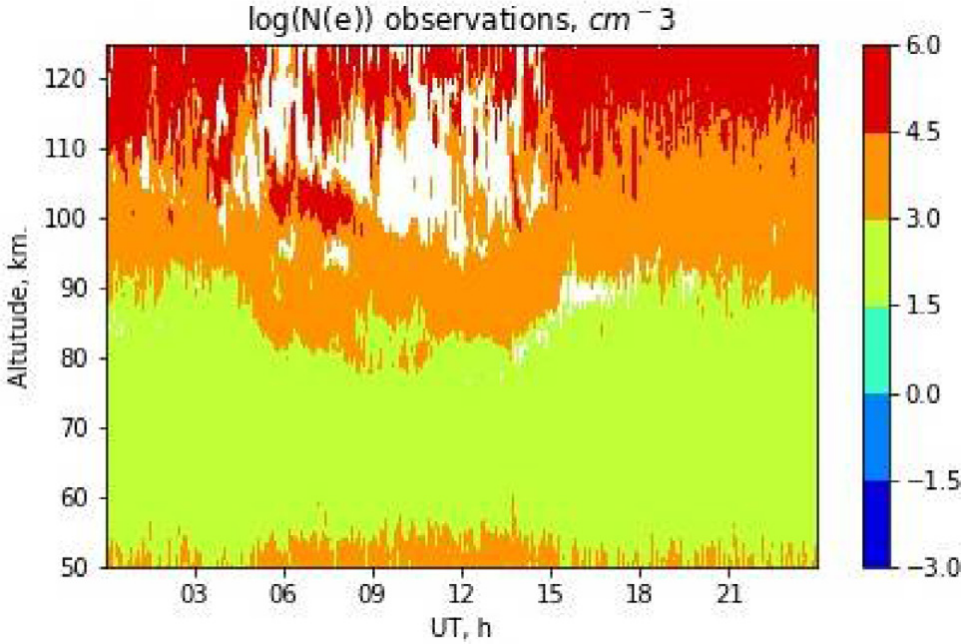
Electron density Ne (logarithm) distribution measured over Tumanny observatory during March 13, 2017. White color denotes values exceeding the largest value of the color-bar.

Each data-file has the following file format (see Fig. 1). Top lines, these are escaped with the ‘#’ sign, form a header of a data-file. A header may have arbitrary number of lines. It provides information on (a) the data holder, (b) the type of the data, and (c) the person responsible for data the production. The type of the data corresponds either to the observations or model results. The last line of a header (which is escaped with the ‘##’ literals) provides a textual ‘helper’ to parse the next line, i.e., the first line of the data-section. It provides the date of the observation in the ‘YYYY-MM-DD’ format, where ‘YYYY’ is a year (four digits), ‘MM’ is a month (two digits), and ‘DD’ is a day number in the month (two digits). Next lines form the tabulated data-table. The first row in this data-table provides a ‘helper’ label, followed by altitudes (in [km]) separated with spaces. Altitude labels should be treated as columns headings. The first column of a data-table provides timestamps (universal time) in the ‘hh:mm’ format. Here ‘hh’ denotes an hour (two digits with leading zeroes if requires), ‘mm’ denotes minutes (also two digits). Timestamp labels should be treated as rows headings, i.e. one line (or one row) represents a single vertical electron density profile at a given moment of time. All values are separated with spaces as the delimiter. A graphical illustration of D-Region electron density Ne variations over Tumanny observatory is presented in Fig. 2. Note that Figure 2 displays logarithm of the electron density variation but not the electron density itself. Such representation is chosen because the plot contains both day-time and night-time observations.

**Fig. 3:**
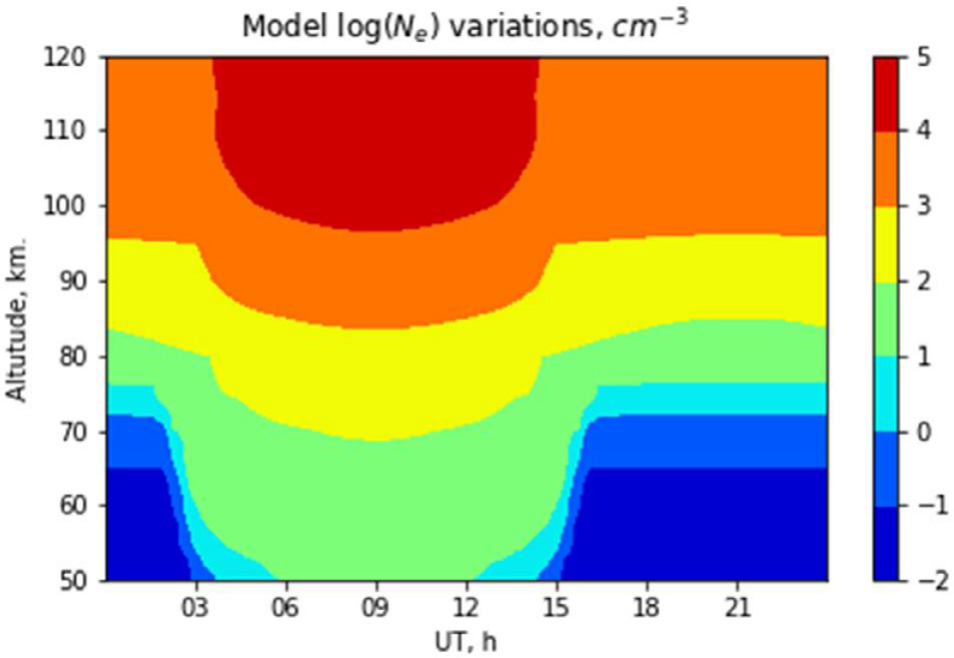
PGI's D-Region model electron density Ne (logarithm) distributions over Tumanny observatory during March 13, 2017.

The ‘model’ directory contains numerically simulated electron density Ne distributions. It follows the same structure, file naming conventions, and very similar data-file format as the described above partial reflection radar data-files. In addition to the label of a date, the first line of the data-section provides longitude (in degrees), latitude (in degrees), Solar radio flux F10.7 index, and kp index values used for simulations. A graphical illustration of D-Region model electron density (logarithm) Ne simulations is presented in Fig. 3. All routines (program codes) that are required to load and plot the data we provide as Python code snippets within the Jupyter notebook (‘example.ipynb’ file).

## 2 Experimental Design, Materials, and Methods

Properties of the Earth's ionosphere D-Region (50 – 90 km) influence on the radio waves propagation (at VLF – HF bands). Despite the long-term history of the ionosphere studies, the D-Region remains one of the most poorly observed and modelled. The proposed dataset provides both the observed and simulated electron density distributions over Tumanny observatory during March 1-31, 2017. This period contains geomagnetically quiet (index kp < 4) and disturbed days. Modelling of quiet-time D-Region variations were of special interest in the related research article [1].

D-Region electron density observations were performed using the partial reflection radar located at Tumanny radio-observatory (69.0°N, 35.7°E). It is the vertical sounding medium-frequency (MF) radar that was designed at Polar Geophysical Institute and put into operations in 1991. A description of the radar facilities was presented, e.g., in [8]. The radar operates on the regular basis till nowadays. The radar quick-views are published by the PGI's stuff on a regular basis at https://pgi.ru/radar2/ . Quick-views are low-resolution images (graphical files) that allow to get an idea what the observations are. They are also useful as a catalog of available observations. Full data sets are not published with quick-views. The interested party is expected to send a request to Aleksander D. Gomonov (Head of Sector of Radiophysical Researches of the Lower Ionosphere, e-mail: gomonov@pgi.ru). The data set is provided on cooperative basis (including, but not limited to, joint research projects, etc.). It is generally free of charge for non-commercial and research purposes. Commercial use is not allowed without signing an agreement. Provided with this article data-files are covered by the same license as this article itself, i.e., the CC BY 4.0 (https://creativecommons.org/licenses/by/4.0/).

Provided with this paper electron density values are derived from ordinary (o-) and extraordinary (x-) waves measurements according to the well-known method (see, e.g., pages 218 - 220 in [9]). This method performs well for altitudes in range of 60 km - 90 km. Outside this range electron density values are subject to uncertain biases.

Numerical simulations of electron density Ne variations were performed with the Polar Geophysical Institute's quiet-time D-Region ionosphere model. A comprehensive description of this theoretical model was published in [10]. Because the model was designed for quiet geomagnetic conditions, Gomonov and co-authors [1] modelled quiet-time Ne variations only. Dates with the index kp < 4 were accounted for, i.e., March 13-14, 16-20, and 24-26. Paper [1] used an averaged D-Region electron density behaviour as the measure of an agreement between the modelled and observed electron density variations. Averages were calculated using the standard formula. Provided with the Jupyter notebook Python code snippets rely on the default NumPy implementation of the ‘average’ function.

## Limitations

• The radar performs well for altitudes in range of 60 km - 90 km. Outside this range electron density values are subject to uncertain biases.
• Radar observations are reliable for all levels of solar activity including severe solar activity periods.
• The radar observes D-Region of the Earth's ionosphere which is too low for typical ionosonde to observe.
• Polar Geophysical Institute's D-Region ionosphere model [10] was designed for quiet geomagnetic conditions.

## Declaration of Competing Interest

The authors declare that they have no known competing financial interests or personal relationships which have, or could be perceived to have, influenced the work reported in this article.

## Appendix Supplementary materials

Image, application 1
